# Bulk-solvent and overall scaling revisited: faster calculations, improved results

**DOI:** 10.1107/S0907444913000462

**Published:** 2013-03-14

**Authors:** P. V. Afonine, R. W. Grosse-Kunstleve, P. D. Adams, A. Urzhumtsev

**Affiliations:** a Lawrence Berkeley National Laboratory, One Cyclotron Road, MS64R0121, Berkeley, CA 94720, USA; b Department of Bioengineering, University of California, Berkeley, Berkeley, CA 94720, USA; c IGBMC, CNRS–INSERM–UdS, 1 Rue Laurent Fries, BP 10142, 67404 Illkirch, France; d Université de Lorraine: Département de Physique – Nancy 1, BP 239, Faculté des Sciences et des Technologies, 54506 Vandoeuvre-lès-Nancy, France

**Keywords:** bulk solvent, scaling, anisotropy, structure refinement, *PHENIX*

## Abstract

A fast and robust method for determining the parameters for a flat (mask-based) bulk-solvent model and overall scaling in macromolecular crystallographic structure refinement and other related calculations is described. This method uses analytical expressions for the determination of optimal values for various scale factors. The new approach was tested using nearly all entries in the PDB for which experimental structure factors are available. In general, the resulting *R* factors are improved compared with previously implemented approaches. In addition, the new procedure is two orders of magnitude faster, which has a significant impact on the overall runtime of refinement and other applications. An alternative function is also proposed for scaling the bulk-solvent model and it is shown that it outperforms the conventional exponential function. Similarly, alternative methods are presented for anisotropic scaling and their performance is analyzed. All methods are implemented in the *Computational Crystallo­graphy Toolbox* (*cctbx*) and are used in *PHENIX* programs.

## Abstract 2

A fast analytical method for calculating mask-based bulk-solvent scale factors and overall anisotropic correction factors is introduced.

## 1. Introduction

Macromolecular crystals typically contain a substantial amount of disordered solvent, ranging from approximately 20 to 90% of the crystal volume, with a mean of 55%, in the Protein Data Bank (PDB; Bernstein *et al.*, 1977 ▶; Berman *et al.*, 2000 ▶). Anisotropy in the diffracted intensities is another common feature of macromolecular crystals that arises from various sources including crystal lattice vibrations (Shakked, 1983 ▶; Sheriff & Hendrickson, 1987 ▶). When modelling diffracted intensities, for example in structure refinement or automated model building, it is therefore critical to account for these two phenomena (see, for example, Jiang & Brünger, 1994 ▶; Urzhumtsev & Podjarny, 1995 ▶; Kostrewa, 1997 ▶; Badger, 1997 ▶; Urzhumtsev, 2000 ▶; Fokine & Urzhumtsev, 2002*a* ▶; Fenn *et al.*, 2010 ▶). The flat bulk-solvent model (Phillips, 1980 ▶; Jiang & Brünger, 1994 ▶) combined with overall anisotropic scaling in either exponential (Sheriff & Hendrickson, 1987 ▶) or polynomial (Usón *et al.*, 1999 ▶) forms is a well established and computationally efficient approach. Alternatives have been proposed (Tronrud, 1997 ▶; Vassylyev *et al.*, 2007 ▶), but are not currently in wide use.

In the commonly used approach, the total structure factor is defined as
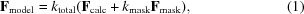
where *k*_total_ is the overall Miller-index-dependent scale factor, **F**_calc_ and **F**_mask_ are the structure factors computed from the atomic model and the bulk-solvent mask, respectively, and *k*_mask_ is a bulk-solvent scale factor. The mask can be computed efficiently using exact asymmetric units as described in Grosse-Kunstleve *et al.* (2011 ▶).

The overall scale factor *k*_total_ can be thought of as the product
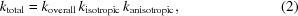
where *k*_overall_ is the overall scale factor and *k*_isotropic_ and *k*_anisotropic_ are the isotropic and anisotropic scale factors, respectively.

*k*_overall_ is a scalar number that can be obtained by minimizing the least-squares residual
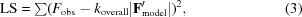
where *F*_obs_ are the observed structure factors and
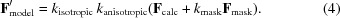
The sum is over all reflections. Solving ∂LS/∂*k*_overall_ = 0 leads to 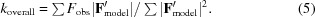
In the exponential model the anisotropic scale factor is defined as
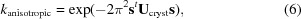
where **U**_cryst_ is the overall anisotropic scale matrix equivalent to **U** ^*^ defined in Grosse-Kunstleve & Adams (2002 ▶); **s** *^t^* = (*h*, *k*, *l*) is the transpose of the Miller-index column vector **s**.

Usón *et al.* (1999 ▶) define a polynomial anisotropic scaling function that can be rewritten in matrix notation as follows:
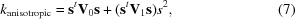
where **V**_0_ and **V**_1_ are symmetric 3 × 3 matrices, *s* ^2^ = **s** *^t^* **G** ^*^ **s** and **G** ^*^ is the reciprocal-space metric tensor. Expression (7) is equivalent to the first terms in the Taylor series expansion of the exponential function (6),
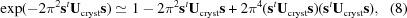
with the constant term omitted. The omission of the constant 1 means that *k*_anisotropic_ is equal to zero for the reflection **F**_000_, as follows from (7). Therefore, in this work we modify (7) by adding the constant 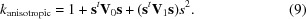
The bulk-solvent scale factor is traditionally defined as
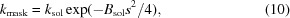
where *k*_sol_ and *B*_sol_ are the flat bulk-solvent model parameters (Phillips, 1980 ▶; Jiang & Brünger, 1994 ▶; Fokine & Urzhumtsev, 2002*b* ▶).

Depending on the calculation protocol, *k*_isotropic_ may be assumed to be a part of *k*_anisotropic_ or it can be assumed to be exponential: *k*_isotropic_ = exp(−*Bs* ^2^/4), where *B* is a scalar parameter. Alternatively, it may be determined as described in §[Sec sec2.3]2.3 below.

The determination of the anisotropic scaling parameters (**U**_cryst_ or **V**_0_ and **V**_1_) and the bulk-solvent parameters *k*_sol_ and *B*_sol_ requires the minimization of the target function (3) with respect to these parameters. Despite the apparent simplicity, this task is quite involved owing to a number of numerical issues (Fokine & Urzhumtsev, 2002*b* ▶; Afonine *et al.*, 2005*a* ▶). Previously, we have developed a robust and thorough procedure (Afonine *et al.*, 2005*a* ▶) to address these issues. This procedure is used routinely in *PHENIX* (Adams *et al.*, 2010 ▶). However, owing to its thoroughness the procedure is relatively slow and may account for a significant fraction of the execution time of certain *PHENIX* applications (for example, *phenix.refine*).

In this paper, we describe a new procedure which is approximately two orders of magnitude faster than the approach described in Afonine *et al.* (2005*a* ▶) and often leads to a better fit of the experimental data. The speed gain is the result of an analytical determination of the optimal bulk-solvent and scaling parameters. The better fit to the experimental data is partially the result of employing a more detailed model for *k*_mask_ compared with the exponential model in equation (10) and is partially a consequence of the new analytical optimization method. Analytical optimization eliminates the possibility of becoming trapped in local minima, which exists in all iterative local optimization methods, including the procedure used previously.

## 2. Methods

### 2.1. Anisotropic scaling: exponential model

To obtain the elements of the anisotropic scaling matrix (6), the minimization of (3) is replaced by the minimization of
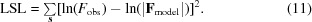
For this, we assume that *F*_obs_ and |**F**_model_| are positive. We also assume that the minima of (3) and (11) are at similar locations. This assumption is not obvious and, as discussed below, may not always hold (see §[Sec sec3.3]3.3 and Table 2). Expression (11) can be rewritten as 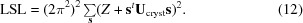
Here, *Z* = [1/(2π^2^)]ln[*F*_obs_(*k*_overall_ *k*_isotropic_|**F**_calc_ + *k*_mask_ **F**_mask_|)^−1^]. Defining
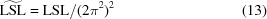
and using
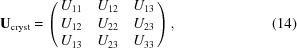
the target function determining the optimal **U**_cryst_ is 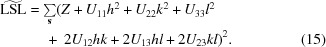
The **U**_cryst_ values that minimize (15) are determined from the condition 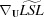 = 0, which gives a system of six linear equations
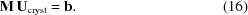
where **M** = 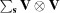, **V** = (*h* ^2^, *k* ^2^, *l* ^2^, 2*hk*, 2*hl*, 2*kl*)^*t*^, ⊗ denotes the outer product and **b** = 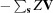.

The desired **U**_cryst_ matrix is determined by solving the system (16):[Disp-formula fd16]

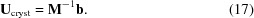



Crystal-system-specific symmetry constraints can be incorporated *via* a constraint matrix (**C**), which we derive from first principles by solving the system of linear equations **R** ^*t*^ **UR** = **U** for all rotation matrices **R** of the crystal-system point group. Alternatively, symmetry constraints are often derived manually and tabulated (Nye, 1957 ▶; Giacovazzo, 1992 ▶). For example, the constraint matrix for the tetragonal crystal system is
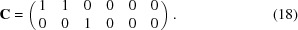
The number of rows in **C** determines the number of independent coefficients of **U**_cryst_. Let **U**_ind_ be the column vector of independent coefficients; the (redundant) set of six coefficients **U**_cryst_ is then obtained *via*

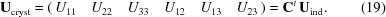
The constraint matrix **C** is introduced into equations (16) and (17) above as follows:
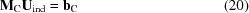
with **M**_C_ = 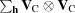, **V**_C_ = **CV**, **b**_C_ = 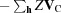 and
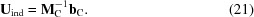
The full **U**_cryst_ is then determined *via* equation (19).

### 2.2. Anisotropic scaling: polynomial model

The polynomial model (Usón *et al.*, 1999 ▶) for anisotropic scaling allows the direct use of the residual (3) to find the optimal coefficients for **V**_0_ and **V**_1_ in equation (9). An advantage of this model is that no assumptions about the similarity of the location of the minima of targets (3) and (11) are required. Conceptually, a disadvantage of equation (9) is that it is only an approximation of equation (6), as was shown above. However, the number of parameters is doubled in equation (9) compared with equation (6), since **V**_0_ and **V**_1_ are treated independently. The increased number of degrees of freedom may therefore compensate for approximation in­accuracies.

Similarly to §[Sec sec2.1]2.1, the optimal coefficients for **V**_0_ and **V**_1_ are determined by the condition ∇**_V_**LS = 0 and can be obtained by solving a system of 12 linear equations. We follow the arguments of Usón *et al.* (1999 ▶) for not using symmetry constraints in this case.

### 2.3. Bulk-solvent parameters and overall isotropic scaling

Defining *K* = *k* ^−2^_total_ = (*k*_overall_ *k*_isotropic_ *k*_anisotropic_)^−2^, the determination of the desired scaling parameters *k*_isotropic_ and *k*_mask_ is reduced to minimizing
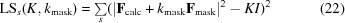
in resolution bins, where *k*_overall_ and *k*_anisotropic_ are fixed. This minimization problem is generally highly overdetermined because the number of reflections per bin is usually much larger than two.

Introducing *w* = |**F**_mask_|^2^, *v* = (**F**_calc_, **F**_mask_) and *u* = |**F**_calc_|^2^ and substitution into (22) leads to 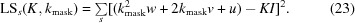
Minimizing (23) with respect to *K* and *k*_mask_ leads to a system of two equations:
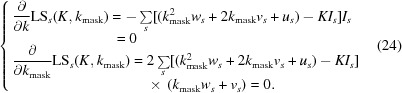
Developing these equations with respect to *k*_mask_,
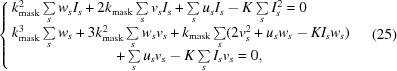
and introducing new notations for the coefficients, we obtain 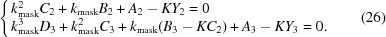
Multiplying the second equation by *Y*_2_ and substituting *KY*_2_ from the first equation into the new second equation, we obtain a cubic equation
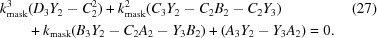
The senior coefficient in (27) satisfies the Cauchy–Schwarz inequality:
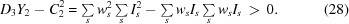
Therefore, equation (27) can be rewritten as
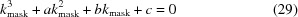
and solved using a standard procedure.

The corresponding values of *K* are obtained by substituting the roots of equation (29) into the first equation in (26):
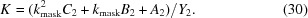
If no positive root exists *k*_mask_ is assigned a zero value, which implies the absence of a bulk-solvent contribution. If several roots with *k*_mask_ ≥ 0 exist then the one that gives the smallest value of LS_*s*_(*K*, *k*_mask_) is selected.

If desired, one can fit the right-hand side of expression (10) to the array of *k*_mask_ values by minimizing the residual
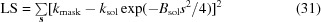
for all *k*_mask_ > 0. This can be achieved analytically as described in Appendix *A* [App appa]. Similarly, one can fit *k*_overall_exp(−*B*_overall_ *s* ^2^/4) to the array of *K* values.

### 2.4. Presence of twinning

In case of twinning with *N* twin-related domains, the total model intensity is
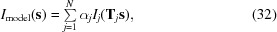
where α*_j_* is the twin fraction of the *j*th domain, **T** *_j_* is the corresponding twin operator (a 3 × 3 rotation matrix) and
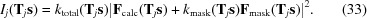

*k*_total_ includes all scale factors (overall, isotropic and anisotropic). We make the reasonable assumption that *k*_total_ and *k*_mask_ are identical for all twin domains.

Finding the twin fractions α*_j_* can be achieved by solving the minimization problem
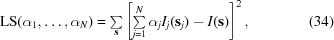
with the constraint condition
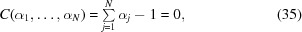
where *I*(**s**) = *F* ^2^_obs_ and **s**_*j*_ = **T**_*j*_ **s**. This constrained minimization problem can be reformulated as an unconstrained minimization problem by the standard technique of introducing a Lagrange multiplier:
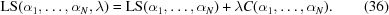
The values {α_1_, …, α_*N*_, λ} that minimize (36) are the solution of the system of *N* + 1 linear equations with *N* + 1 variables:
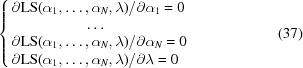
or
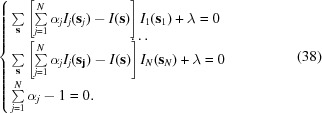
The solution of this system is
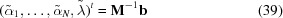
with the (*N* + 1) × (*N* + 1) matrix
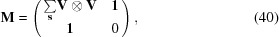
and
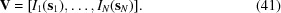
Here, **1** is a row or column containing *N* unit elements to complete the matrix **M** and
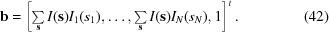
The values of ∊ are expected to be between 0 and 1, and λ is proportional to the sum of squared intensities. Therefore, it is numerically beneficial to multiply the λ*C*(α_1_, …, α_*N*_) term in (36) by a constant 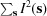 in order to make the value for λ numerically similar to the values for the twin fractions α. 

Once the twin fractions have been found, the procedure described in §[Sec sec2.3]2.3 can be used to obtain the overall and bulk-solvent scale factors. Similarly to (23), we can write
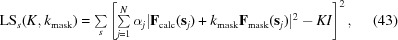
where α*_j_* are known twin fractions and *K* and *k*_mask_ are the scale factors to be determined. Similarly to §[Sec sec2.3]2.3, we obtain 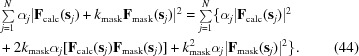
Introducing new variables as before for equation (23) leads to
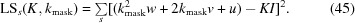
The determination of the twin fractions α and scales *k*_total_ and *k*_mask_ are iterated several times until convergence. The determination of α does not guarantee that the individual twin fractions α_*j*_ are in the range 0–1. For any α_*j*_ outside this range the corresponding twin operation is ignored for the current iteration and the new smaller set of twin fractions and scales are redetermined. However, in the next iteration the full set of α is tried again.

## 3. Results

### 3.1. Implementation of the new protocol

The scale factors involved in the calculation of **F**_model_ according to equation (1) are highly correlated. Therefore, the order of their determination is important. Empirically, we found that the determination of *k*_isotropic_ and *k*_mask_ followed by the determination of *k*_anisotropic_ works optimally in most cases. The determination of (*k*_mask_, *k*_isotropic_) and *k*_anisotropic_ is re­peated several times until the *R* factor decreases by less than 0.01% between cycles. The number of cycles required to reach convergence is typically between 1 and 5.

To determine *k*_anisotropic_, our protocol can make use of three available scaling methods: polynomial (poly; §[Sec sec2.2]2.2), exponential with analytical calculation of the optimal parameters (exp_anal_; §[Sec sec2.1]2.1) and exponential with the optimal parameters obtained *via* L-BFGS (Liu & Nocedal, 1989 ▶) minimization (exp_min_; Afonine *et al.*, 2005*a* ▶). The three methods can be tested independently, in which case the result with the lowest *R* factor is accepted. However, because exp_min_ is up to an order of magnitude slower than the other two methods it is not expected to be used routinely.

**Figure 1:**
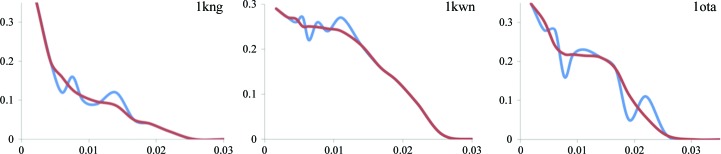
Examples of smoothening of *k*_mask_. The original *k*_mask_ (blue; obtained as the solution of equation 29) and that after smoothening (red) are shown for three PDB entries with the PDB codes shown on the plots.

The calculation of *k*_isotropic_ and *k*_mask_ requires dividing the data into resolution bins (§[Sec sec3.2]3.2). If oscillation of *k*_mask_ between bins occurs, smoothening (Savitzky & Golay, 1964 ▶) is applied to the bin-wise determined values of *k*_mask_ such that it reduces the oscillations without altering the monotonic behavior of *k*_mask_ as a function of resolution (see Fig. 1 ▶). Finally, the smoothed values are assigned to individual reflections using linear interpolation. The *k*_isotropic_ scales are updated using equation (5) in order to account for the changed *k*_mask_.

As illustrated in §[Sec sec3.2]3.2, the minimum of the *R*-factor function
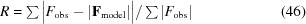
and the minimum of the least-squares function (22) can be at significantly different locations in the (*k*_mask_, *k*_isotropic_) parameter space. To assure that the final (*k*_mask_, *k*_isotropic_) values correspond to the lowest *R* factor, a fast grid search is performed around the optimal values of the least-squares function.

### 3.2. Binning

The goal of binning is to group data by common features to characterize each group by a set of common parameters. Here, the key parameter is the resolution *d* of reflections. Binning schemes with bins containing an approximately equal number of reflections (*i.e.* the resolution range is uniformly sampled in *d* ^−3^) or a predefined number of bins are typically used. Since the low-resolution region of the data is sparse, such binning schemes tend to produce only one or very few low-resolution bins, which is insufficient to best model the bulk-solvent contribution. Unfortunately, decreasing the number of reflections per bin will disproportionally increase the number of bins (*N*_bins_) at higher resolution and may still provide insufficient detail for the low-resolution data (Table 1 ▶).

**Figure 2:**
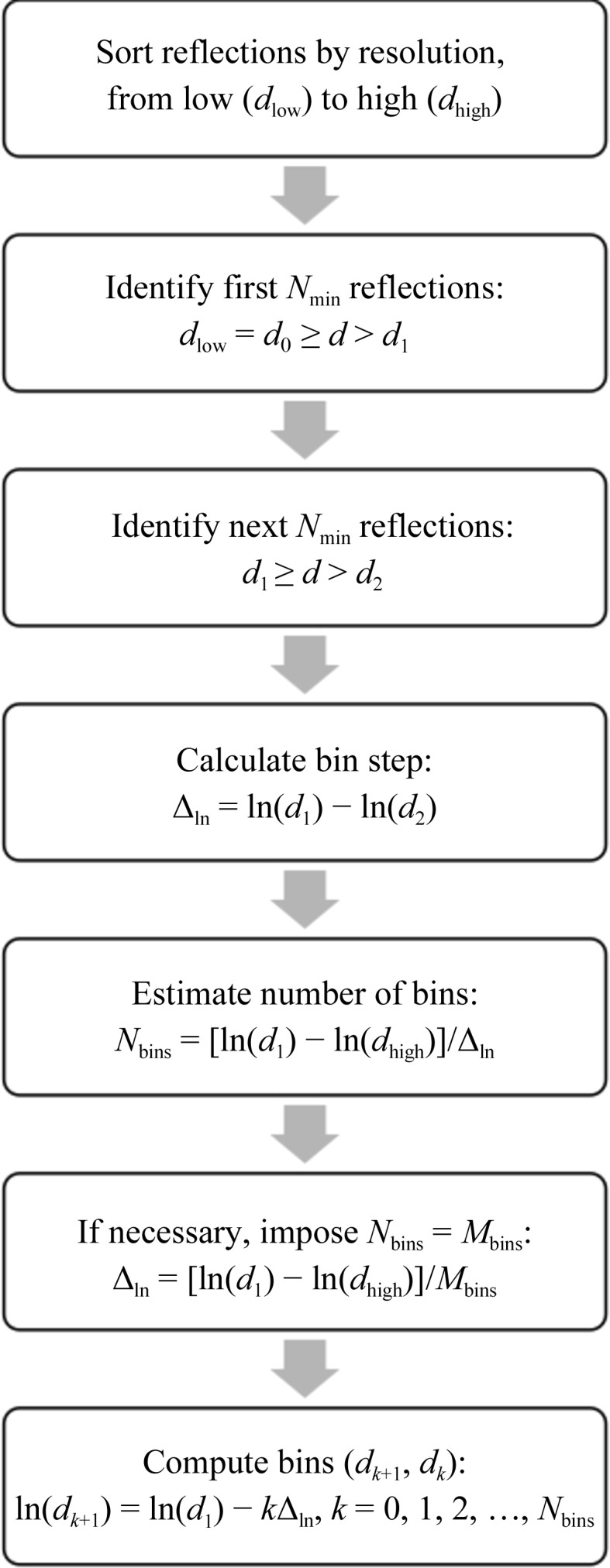
Flowchart of the logarithmic resolution-binning algorithm.

An alternative approach which divides the resolution range uniformly on a logarithmic scale ln(*d*) (Urzhumtsev *et al.*, 2009 ▶) efficiently solves this problem. The flowchart of the algorithm is shown in Fig. 2 ▶. This scheme allows the higher resolution bins to contain more reflections than the lower resolution bins and more detailed binning at low resolution without increasing the total number of bins. An additional reason for using logarithmic binning is that the dependence of the scales on resolution is approximately exponential (see previous sections), which makes the variation of scale factors more uniform between bins when a logarithmic binning algorithm is used. Table 1 ▶ compares binning performed uniformly in *d* ^−3^ and in ln(*d*) spacing for three data sets (PDB entries 3hay, 1kwn and 3gk8). Note the data completeness of the low-resolution bins.

### 3.3. Systematic tests

We evaluated the performance of the new scaling protocol by applying it to approximately 40 000 data sets selected from the PDB. The structures were selected by evaluating all PDB entries using *phenix.model_vs_data* (Afonine *et al.*, 2010 ▶) and excluding all entries for which the recalculated *R*_work_ was greater than the published value by five percentage points.

To score the test results three crystallographic *R* factors (46) were computed using all reflections, using only low-resolution reflections and using only high-resolution reflections. Low-resolution reflections were selected using the condition *d*_min_ > 8 Å but selecting at least the 500 lowest resolution reflections. High-resolution reflections were taken from the highest resolution bin. Each of the three anisotropic scaling methods (poly, exp_anal_ and exp_min_) was tested independently within each run. Additionally, two other tests were performed: one combining poly and exp_anal_ as described in §[Sec sec3.1]3.1 (referred to as poly+exp_anal_) and the other using the protocol of Afonine *et al.* (2005*a* ▶) (referred to as old).

**Figure 3:**
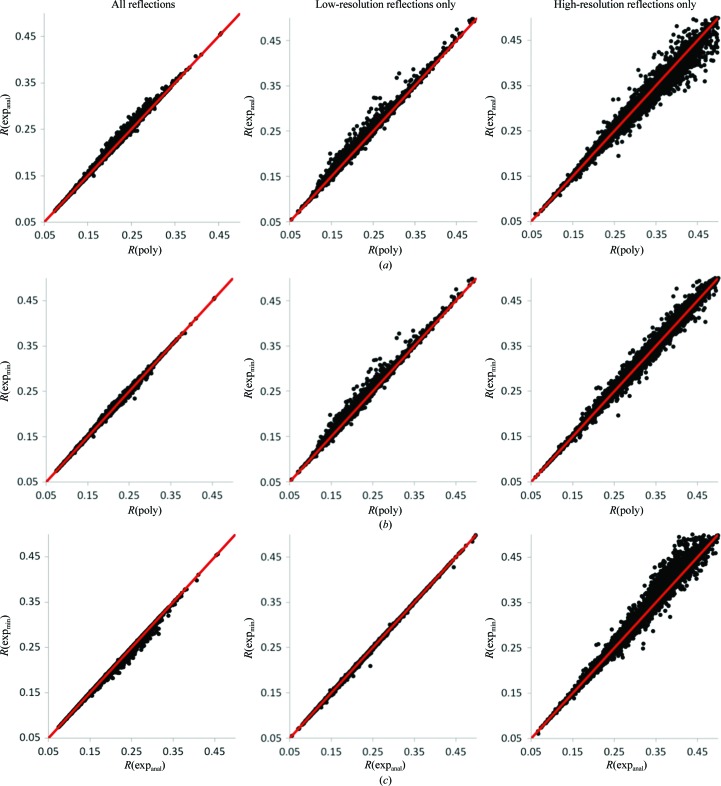
A comparison of the new scaling protocol using different models for the anisotropic scale factor. *R versus R* factor scatter plots for (*a*) poly *versus* exp_anal_, (*b*) poly *versus* exp_min_ and (*c*) exp_anal_ *versus* exp_min_ *R* factors were computed using all reflections (left), low-resolution reflections only (middle) and high-resolution reflections only (right). See §[Sec sec3.3]3.3 for details.

**Figure 4:**
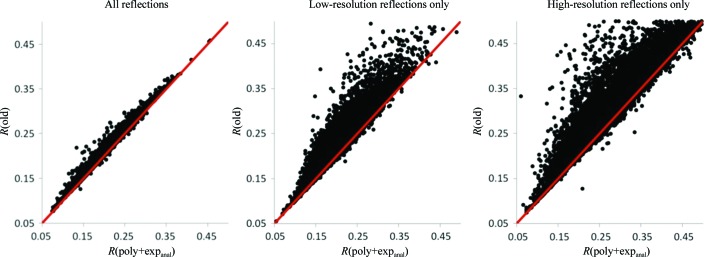
*R* *versus* *R* factor scatter plots comparing the new scaling protocol using poly+exp_anal_ for the anisotropic scale factor with the old protocol. For each structure the full set of structure factors available from the PDB was used to calculate scale factors and to calculate *R* factors (left). Using the same scale-factor values the *R* factors were calculated separately for the low-resolution reflections (middle) and high-resolution reflections (right). A large spread of points in the vertical direction above the diagonal (red line) in these latter plots indicates that in many cases the scale factors produced by the old protocol resulted in a poorer fit to the data at low and high resolutions, while the new protocol generates scale factors with a good fit across all resolution ranges. See §[Sec sec3.3]3.3 for details.

Fig. 3 ▶ shows a comparison of the alternative methods for determining *k*_anisotropic_ (see §[Sec sec3.1]3.1). Comparing the polynomial model (poly) *versus* the analytical exponential model (exp_anal_), with a few minor exceptions poly results in slightly lower *R* factors overall and for the low-resolution reflections, while exp_anal_ results in lower *R* factors for the high-resolution reflections. Comparing poly *versus* the original exponential model using minimization (exp_min_), the *R* factors are very similar overall and for the high-resolution reflections, while poly often results in lower *R* factors for the low-resolution reflections. Comparing the two different exponential models, exp_min_ results in lower *R* factors overall and nearly identical results for low-resolution reflections, but exp_anal_ results in lower *R* factors for the high-resolution reflections. Fig. 4 ▶ compares the new protocol combining poly and exp_anal_ with the old protocol. With very few exceptions, the new protocol performs better for all three resolution groups.

**Figure 5:**
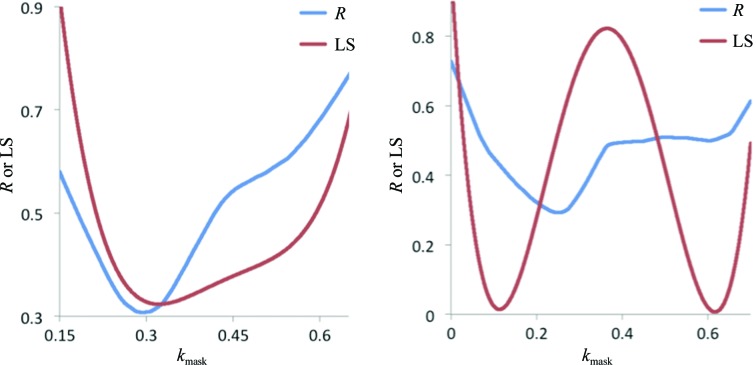
Plots of *R* factors (with *k*_isotropic_ = 0.0961) and the LS function (with *k*_isotropic_ = 0.0863) for PDB entry 1kwn (left) and *R* factors (with *k*_isotropic_ = 0.0131) and the LS function (with *k*_isotropic_ = 0.0151) for PDB entry 1hqw (right), illustrating that the minima of the *R*-factor function (46) and the LS function (22) can be at significantly different locations in parameter space. In such cases, a line search around the value of *k*_mask_ obtained by minimization of the LS function is necessary in order to obtain a value that minimizes the *R* factor. For plotting purposes, the values of the LS function were scaled to be similar to the *R* factors.

As described above, occasionally the minima of the *R*-factor function (46) and the LS function (22) are at significantly different locations in the (*k*_mask_, *k*_isotropic_) parameter space (see Fig. 5 ▶). For example, considering *k*_isotropic_ to be a single-value scalar the pair (*k*_mask_, *k*_isotropic_) that minimizes the *R* factor in the low-resolution range of PDB data set 1kwn is (0.2913, 0.0961), while the pair (0.3218, 0.0863) minimizes the LS function. The corresponding *R* factors are 0.3073 and 0.3372, respectively. The data for PDB entry 1hqw lead to an even more dramatic difference, in which the pairs (*k*_mask_, *k*_isotropic_) that minimize the *R* factor and the LS function are (0.25, 0.0131) and (0.6166, 0.0151), respectively, and the corresponding *R* factors are 0.2924 and 0.5046. We made a similar observation for the overall anisotropic scale *k*_anisotropic_, as illustrated in Table 2 ▶. For this, the best values for **U**_cryst_ were determined *via* a systematic search for the minima of the functions (3), (11) and (46) for three combinations of structures and high-resolution cutoffs. Note the difference in the optimal **U**_cryst_ values and the corresponding *R* factors.

**Figure 6:**
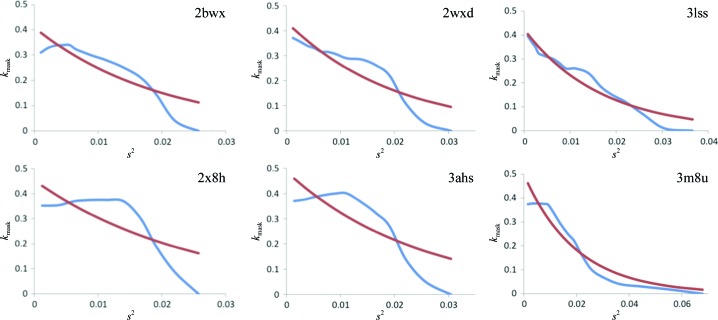
Plots of *k*_mask_ as a function of resolution (*s* ^2^) for six selected PDB entries. The blue lines show *k*_mask_ as determined using the new method. The red lines show *k*_mask_ based on the exponential function (10) using optimized *k*_sol_ and *B*_sol_ parameters.

The parameterization of the total model structure factor (1) does not make any assumption about the shape of *k*_mask_; for example, it does not assume it to be exponential (10). This provides an opportunity to explore the behavior of *k*_mask_ as a function of resolution and compare it with *k*_mask_ obtained *via* (10). Fig. 6 ▶ illustrates the differences between the two methods of determining *k*_mask_ for six representative PDB entries selected from approximately 40 000 entries after inspection of the *k*_mask_ values. We observe that the plots of the values obtained using our new approach are in general significantly different from the exponential function. This observation is in line with Fig. 1 ▶ of Urzhumtsev & Podjarny (1995 ▶).

At very low resolution the structure factors computed from the atomic model are approximately anticorrelated to the structure factors computed from the bulk-solvent mask:
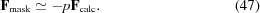
Here, *p* is a scale factor (Urzhumtsev & Podjarny, 1995 ▶). Relation (47) is the basis for alternative bulk-solvent scaling methods that employ the Babinet principle (Moews & Kretsinger, 1975 ▶; Tronrud, 1997 ▶). Substitution of relation (47) into equation (1) yields
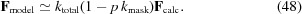
Obviously, **F**_model_ is invariant for any combination of scale factors *k*_total_ and *k*_mask_ satisfying the condition
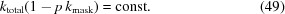
Since our new scaling procedure determines *k*_mask_ and *k*_isotropic_ (which are part of *k*_total_) simultaneously, without imposing constraints on their values, these scale factors may assume unusual values in the low-resolution range. However, we observe that in practice this only happens for a very small number of the test cases.

## 4. Discussion

A new method for overall anisotropic and bulk-solvent scaling of macromolecular crystallographic diffraction data has been developed which is an improvement over the existing algorithm of flat (mask-based) bulk-solvent modeling and overall anisotropic scaling, versions of which are routinely used in various refinement packages such as *CNS* (Brunger, 2007 ▶), *REFMAC* (Murshudov *et al.*, 2011 ▶) and *phenix.refine* (Afonine *et al.*, 2012 ▶). In the process of developing this method, we concluded that the bulk-solvent scale factor *k*_mask_ deviates quite significantly from the exponential model that has traditionally been used. This new method is approximately two orders of magnitude faster than the previous implementation and yields similar or often better *R* factors. Table 3 ▶ compares runtimes for a number of selected cases covering a broad range of resolutions and atomic model sizes. Therefore, the computational speed of the new method makes it possible to robustly compute bulk-solvent and anisotropic scaling parameters even as part of semi-interactive procedures.

An inherent feature of the mask-based bulk-solvent model is that it relies on the existing atomic model to compute the mask. This in turn implies that any unmodeled (as atoms) parts of the unit cell are considered to belong to the bulk-solvent region. This may obscure weakly pronounced features in residual maps such as partially occupied solvent or ligands. This is common to all mask-based bulk-solvent modeling methods, leading to the development of algorithms to account for missing atoms (Roversi *et al.*, 2000 ▶). In the future, improved maps may be obtained by combining this latter approach with the new fast overall anisotropic and bulk-solvent scaling method that we have presented.

The new method is implemented in the *cctbx* project (Grosse-Kunstleve *et al.*, 2002 ▶) and is used in a number of *PHENIX* applications since v.1.8 of the software, most notably *phenix.refine* (Afonine *et al.*, 2005*b* ▶, 2012 ▶), *phenix.maps* and *phenix.model_vs_data* (Afonine *et al.*, 2010 ▶). The *cctbx* project is available at http://cctbx.sourceforge.net under an open-source license. The *PHENIX* software is available at http://www.phenix-online.org.

All results presented are based on *PHENIX* v.1.8.1.

## Figures and Tables

**Table 1:** Comparison of binning schemes performed with *d* ^−3^ and ln(*d*) spacing for three selected PDB data sets: 1kwn, 3hay and 3gk8 All three data sets have very low completeness in the lowest resolution bin, which *d* ^−3^ binning obscures while ln(*d*) binning makes clear even when using approximately half the number of bins. Completeness in the high-resolution region is similar in the two binning schemes. For each binning method three columns of data are presented: resolution range (Å), completeness and number of reflections.

	1kwn		3hay		3gk8	
Bin No.	*d* ^−3^	ln(*d*)	*d* ^−3^	ln(*d*)	*d* ^−3^	ln(*d*)
1	19.96–3.25 0.967 4363	19.96–7.87 0.860 301	44.86–13.44 0.932 715	44.86–17.61 0.852 300	22.18–5.00 0.906 1938	22.18–8.16 0.610 300
2	3.25–2.58 0.997 4280	7.87–6.33 0.971 300	13.43–10.71 1.000 716	17.58–14.23 1.000 301	5.00–3.98 0.994 2052	8.15–7.00 0.993 300
3	2.58–2.26 0.999 4214	6.33–5.10 0.966 564	10.71–9.37 1.000 688	14.22–11.51 1.000 556	3.98–3.48 0.997 2060	7.00–6.01 0.996 452
4	2.26–2.05 1.000 4218	5.10–4.10 0.961 1037	9.37–8.52 1.000 693	11.51–9.31 1.000 1011	3.48–3.16 0.995 2051	6.01–5.16 0.994 700
5	2.05–1.90 0.990 4135	4.10–3.30 0.986 1987	8.52–7.91 1.000 679	9.31–7.53 1.000 1853	3.16–2.93 0.976 1988	5.16–4.43 0.993 1087
6	1.90–1.79 0.993 4133	3.30–2.66 0.997 3772	7.91–7.45 1.000 673	7.53–6.10 1.000 3448	2.93–2.76 0.968 1973	4.43–3.81 0.996 1735
7	1.79–1.70 0.992 4119	2.66–2.14 0.999 7177	7.45–7.08 1.000 675	6.10–4.99 0.997 5905	2.76–2.62 0.958 1902	3.81–3.27 0.996 2716
8	1.70–1.63 0.989 4070	2.14–1.72 0.993 13453	7.08–6.77 1.000 657		2.62–2.51 0.952 1961	3.27–2.81 0.979 4149
9	1.63–1.57 0.988 4094	1.72–1.38 0.990 25516	6.77–6.51 1.000 672		2.51–2.41 0.954 1941	2.81–2.41 0.955 6410
10	1.57–1.51 0.990 4093	1.38–1.20 0.989 28106	6.51–6.29 1.000 671		2.41–2.33 0.941 1876	2.41–2.07 0.931 9748
11	1.51–1.46 0.987 4036		6.28–6.09 1.000 657		2.33–2.26 0.933 1897	2.07–1.85 0.827 9681
12	1.46–1.42 0.990 4073		6.09–5.92 1.000 655		2.26–2.19 0.940 1881	
13	1.42–1.39 0.993 4088		5.91–5.76 1.000 666		2.19–2.13 0.931 1876	
14	1.39–1.35 0.992 4057		5.76–5.62 1.000 656		2.13–2.08 0.914 1838	
15	1.35–1.32 0.992 4077		5.62–5.49 1.000 667		2.08–2.03 0.897 1834	
16	1.32–1.29 0.995 4052		5.49–5.38 1.000 653		2.03–1.99 0.891 1766	
17	1.29–1.27 0.991 4047		5.38–5.27 1.000 635		1.99–1.95 0.865 1765	
18	1.27–1.24 0.991 4045		5.27–5.17 1.000 663		1.95–1.92 0.825 1645	
19	1.24–1.22 0.988 4026		5.17–5.08 1.000 660		1.91–1.88 0.767 1537	
20	1.22–1.20 0.972 3993		5.08–4.99 0.973 623		1.88–1.85 0.732 1497	

**Table 2:** Comparison of **U**_cryst_ corresponding to the minima of the functions LS (3), LSL (11) and *R* factor (46) To improve readability, the **U**_cryst_ are shown as *B* values with respect to a Cartesian basis (Grosse-Kunstleve & Adams, 2002 ▶). To reduce the runtimes for the systematic parameter searches (see text), we have selected examples with symmetry constraints leading to all-zero off-diagonal elements.

PDB code	Optimization target	*B*_11_, *B*_22_, *B*_33_	*R* factor
2fih	*R* factor	−2.15, −1.85, −1.60	0.1935
	LS	−4.20, −3.90, −3.35	0.2179
	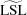	−2.65, −1.95, −1.60	0.1939
2fih (data cut at 2.5 Å)	*R* factor	−9.30, −10.20, −10.35	0.2417
	LS	−18.35, −19.65, −20.75	0.2599
	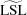	−38.25, −42.15, −46.20	0.3769
1ous (data cut at 6.5 Å)	*R* factor	9.25, −2.20, 4.35	0.2082
	LS	2.90, −2.45, 8.60	0.2086
	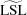	19.55, 6.55, 12.85	0.2088

**Table 3:** Runtime comparison for selected PDB entries Absolute runtimes for the new protocol range from a few hundredths of a second to a second.

PDB code	Resolution (Å)	No. of atoms	No. of reflections	Speed gain
1us0	0.66	3679	511265	105
1akg	1.10	136	4471	132
1ous	1.20	3784	104889	86
1yjp	1.80	66	495	64
1f8t	1.95	3593	28288	104
1av1	4.00	6588	16201	110
1jl4	3.99	4474	7428	78
2i07	4.0	12157	20412	126
2gsz	4.2	16344	17131	166

## Appendix A Analytical derivation of a one-Gaussian approximation of a one-dimensional discrete data set

Our goal is to approximate a set of data points {*Y*(*x*)}*^N^* *_j_* = 1 with a Gaussian function,

For this, we use the standard approach of minimizing a least-squares (LS) function,

If *Y*(*x_j_*) ≥ 0 ∀ *x_j_*, *j* = 1, *N*, the minimization of LS can be replaced by the minimization of

The minimum of this LSL function can be determined analytically,

Defining *u* = ln(*a*), *v_j_* = *x_j_* ^2^, *d_j_* = ln[*Y*(*x_j_*)], we obtain

The variables {*a*, *b*} minimizing the LSL function are determined by the condition

This leads to

and

Defining *p* = , *q* = , *r* =  and *s* = , we obtain

and

From this, we obtain

and finally
