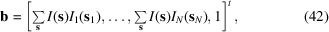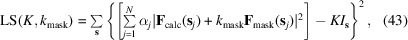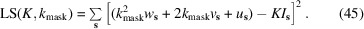# Bulk-solvent and overall scaling revisited: faster calculations, improved results. Corrigendum.

**DOI:** 10.1107/S2059798323004825

**Published:** 2023-06-20

**Authors:** P. V. Afonine, R. W. Grosse-Kunstleve, P. D. Adams, A. Urzhumtsev

**Affiliations:** a Lawrence Berkeley National Laboratory, One Cyclotron Road, MS64R0121, Berkeley, CA 94720, USA; b Department of Bioengineering, University of California Berkeley, Berkeley, CA 94720, USA; c IGBMC, CNRS–INSERM–UdS, 1 Rue Laurent Fries, BP 10142, 67404 Illkirch, France; d Université Nancy: Département de Physique – Nancy 1, BP 239, Faculté des Sciences et des Technologies, 54506 Vandoeuvre-lès-Nancy, France; University of Cambridge, United Kingdom

**Keywords:** *Phenix*, anisotropy, bulk solvent, scaling

## Abstract

Equations in Sections 2.3 and 2.4 of the article by Afonine *et al.* [*Acta Cryst.* (2013). D**69**, 625–634] are corrected.

## Abstract 2

The article by Afonine *et al.* [ *Acta Cryst.* (2013). D**69**, 625–634] is corrected.

In the article by Afonine *et al.* (2013 ▸) some improper notations and errors in several equations in Sections 2.3 and 2.4 have been corrected. We note that the *Computational Crystallography Toolbox* (Grosse-Kunstleve *et al.*, 2002 ▸) has been using the correct version of these equations since 2013. Updated versions of Section 2.3 and equations (42), (43) and (45) are given below.

### 2.3. Bulk-solvent parameters and overall isotropic scaling

Assuming the resolution-dependent scale factors *k*_mask_(**s**) and *k*_isotropic_(**s**) to be constants *k*_mask_ and *k*_isotropic_ in each thin resolution shell, the determination of their values is reduced to minimizing the residual

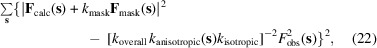

where the sum is calculated over all reflections **s** in the given resolution shell, and *k*_overall_ and *k*_anisotropic_(**s**) are calculated previously and fixed. This minimization problem is generally highly over-determined because the number of reflections per shell is usually much larger than two.

Introducing *w*_**s**_ = |**F**_mask_(**s**)|^2^, 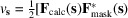 + 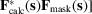 , *u*_**s**_ = |**F**_calc_(**s**)|^2^, 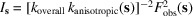 and 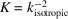 and substituting them into (22) leads to the minimization of

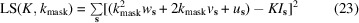

with respect to *K* and *k*_mask_. This leads to a system of two equations:

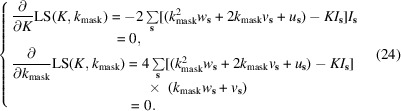

Developing these equations with respect to *k*_mask_,

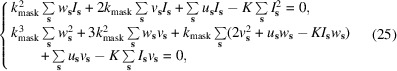

and introducing new notations for the coefficients, we obtain

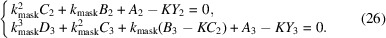

Multiplying the second equation by *Y*_2_ and substituting *KY*_2_ from the first equation into the new second equation, we obtain a cubic equation with fixed coefficients

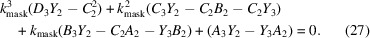

The senior coefficient in equation (27) satisfies the Cauchy–Schwarz inequality:

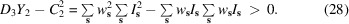

Therefore, equation (27) can be rewritten as

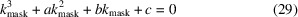

and solved using a standard procedure.

The corresponding values of *K* are obtained by substituting the roots of equation (29) into the first equation in equation (26),

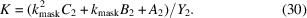

If no positive root exists, *k*_mask_ is assigned a zero value, which implies the absence of a bulk-solvent contribution. If several roots with *k*_mask_ ≥ 0 exist then the one that gives the smallest value of LS(*K*, *k*_mask_) is selected.

If desired, one can fit the right-hand side of expression (10) to the array of *k*_mask_ values by minimizing the residual

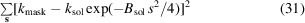

for all *k*_mask_ > 0. This can be achieved analytically as described in Appendix *A*. Similarly, one can fit *k*_overall _exp(−*B*_overall_ *s* ^2^/4) to the array of *K* values.

Equations (42), (43) and (45) in Section 2.4 of Afonine *et al.* (2013 ▸) are also updated as follows